# Language as a Window Into the Altered State of Consciousness Elicited by Psychedelic Drugs

**DOI:** 10.3389/fphar.2022.812227

**Published:** 2022-03-22

**Authors:** Enzo Tagliazucchi

**Affiliations:** ^1^ Latin American Brain Health Institute (BrainLat), Universidad Adolfo Ibanez, Santiago, Chile; ^2^ Departamento de Física, Universidad de Buenos Aires and Instituto de Física de Buenos Aires (IFIBA, CONICET), Pabellón I, Ciudad Universitaria (1428), Buenos Aires, Argentina

**Keywords:** psychedelics, language, consciousness, natural language processing, pharmacology

## Abstract

Psychedelics are drugs capable of eliciting profound alterations in the subjective experience of the users, sometimes with long-lasting consequences. Because of this, psychedelic research tends to focus on human subjects, given their capacity to construct detailed narratives about the contents of their consciousness experiences. In spite of its relevance, the interaction between serotonergic psychedelics and language production is comparatively understudied in the recent literature. This review is focused on two aspects of this interaction: how the acute effects of psychedelic drugs impact on speech organization regardless of its semantic content, and how to characterize the subjective effects of psychedelic drugs by analyzing the semantic content of written retrospective reports. We show that the computational characterization of language production is capable of partially predicting the therapeutic outcome of individual experiences, relate the effects elicited by psychedelics with those associated with other altered states of consciousness, draw comparisons between the psychedelic state and the symptomatology of certain psychiatric disorders, and investigate the neurochemical profile and mechanism of action of different psychedelic drugs. We conclude that researchers studying psychedelics can considerably expand the range of their potential scientific conclusions by analyzing brief interviews obtained before, during and after the acute effects. Finally, we list a series of questions and open problems that should be addressed to further consolidate this approach.

## Introduction

Both humans and other animals display a natural tendency to consume drugs that transiently modify their behavior, cognition and overall state of consciousness (Samorini, 2002). From an evolutionary standpoint, the reasons behind this inclination are not yet fully understood. What is certain is that, throughout history, some humans have adopted an exploratory attitude towards drugs, experiencing their effects and then communicating to others the nature of their subjective experiences by means of written or spoken language (Escohotado, 1999). The urge to report drug-induced experiences is notable for the serotonergic psychedelics (5-HT2A receptor agonists), a class of compounds capable of eliciting deep modifications in the contents of consciousness, including changes to visual, auditory and somatosensory perception, distortions in the sense of self and in the spatial location and extent of body boundaries, increased emotional volatility, and mystical-type experiences, which encompass feelings of bliss, transcendence, ineffability and selflessness, among other defining characteristics (Nichols, 2016; Preller and Vollenweider, 2016; Johnson et al., 2019). Perhaps the most well-known recollection of a drug-induced experience in Western literature, “The Doors of Perception” by Aldous Huxley, stems from the author’s experimentation with mescaline, a naturally occurring psychedelic phenethylamine (Huxley, 1954).

Due to the complexity of the effects they induce, it is almost impossible for users of psychedelic drugs to communicate the nature of their subjective experience without resorting to language or other form of explicit reporting (Shulgin et al., 1986). This is not true for most psychoactive drugs: the effects of sedatives or stimulants, for example, can be directly appreciated by the observation of behavior, which validates the use of animal models for their investigation (Gonçalves, 2021). In contrast, the subjective effects of psychedelics are not linked to rigid and characteristic behavioral patterns, since they can include states of excitation or relaxation depending on the background and intention of the user (“set”), as well as the surrounding environment (“setting”) (Carhart-Harris et al., 2018). In other words, there is nothing in the behavior of an individual under the acute effects of 75 μg of lysergic acid diethylamide (LSD, a potent psychedelic drug) that unequivocally indicates the presence of visual distortions or feelings of body boundlessness - only by explicit linguistic reports is this individual capable of communicating the full extent of the drug-induced subjective effects. While some recurrent behaviors are considered markers of psychedelic drug action in animal models (such as the head twitch) (Hanks and González-Maeso, 2013) the consensus is that human research is necessary to achieve a complete understanding of the neuropharmacology of serotonergic psychedelics (Shulgin et al., 1986; Nichols, 2016).

Language plays a central role in the investigation of serotonergic psychedelics, yet studies of the interaction between these drugs and language production are relatively underrepresented in the literature. Our goal is to review studies supporting the analysis of language as an important window into the subjective effects elicited by psychedelic drugs, their neurochemical mechanisms of action, and their effects on the large-scale brain networks supporting human cognitive function and consciousness. To introduce the need of studying verbal or written reports of subjective experiences, we will first discuss some of the unique methodological problems raised by psychedelic drugs, and briefly mention some of the advantages and limitations of unconstrained natural language reports compared to other tools to quantify subjective experience. Before conducting an exhaustive review of the literature, we will briefly introduce fundamentals of natural language processing (NLP), a set of computational tools used to extract meaningful quantitative information from reports produced by humans (Chowdhury, 2003). Afterwards, we will review studies focusing on the semantic analysis of retrospective reports and the investigation of speech produced under the acute drug effects (as opposed to analyses of acoustic features of speech). After presenting an overview of how these different dimensions of analysis inform important aspects of the psychedelic experience, ranging from phenomenology to the underlying neurochemistry, we will discuss a series of open problems that outline possible new directions for the use of natural language in the study of psychedelics.

## How Should Psychedelic Drugs Be Studied

**FIGURE 1:**
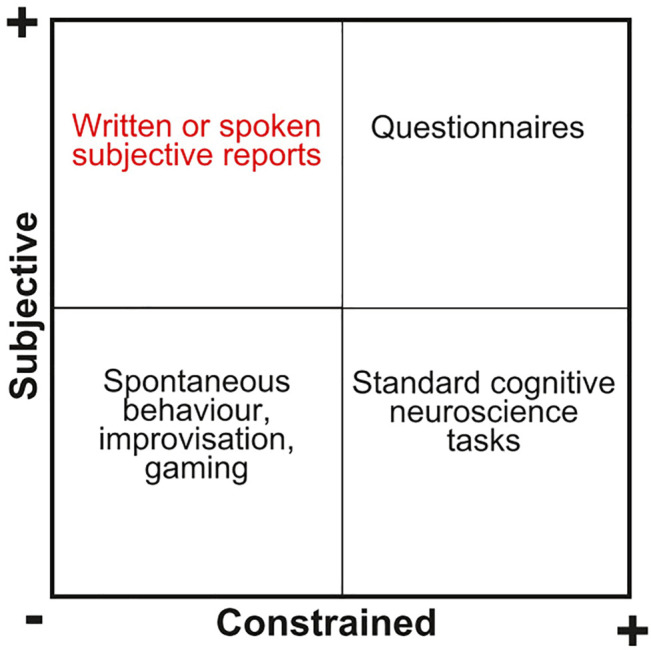
Different ways of approaching the study of the effects of psychedelic drugs. The first dimension (*x* axis) indexes whether the experimental paradigm constrains subjects to adopt a set of predefined behavioral patterns (for instance, when interacting with the interface of a rigidly scripted cognitive experiment). The second dimension (*y* axis) indexes whether the experiment intends to gather information related to the subjective experience of the participants, or to measure their capacity to perform certain tasks or to achieve specific goals. Natural language reports, the main topic of this review, constitute a comparatively unconstrained method to investigate the effects of psychedelic drugs on subjective experience and spontaneous cognitive processing.

The consensus view is that psychedelic research must necessarily include human participants, yet what is the adequate methodology to investigate these participants and their subjective experiences? Figure 1 contains a simple taxonomy of possible experimental approaches towards the study of these compounds. The first dimension (*x* axis) indexes the freedom that the participants have to report their experiences during and after the experiment, while the second (*y* axis) determines whether the experiment is oriented to measure their subjective experience, or to assess their performance in goal-oriented tasks. Ever since the earliest days of psychedelic research, it is clear that certain cognitive functions can be severely impaired during the acute effects of these drugs (Jarvik et al., 1955; Bayne and Carter, 2018). In the case of attention, for instance, this impairment limits the use of the standard approach of cognitive neuroscience (right lower quadrant in Figure 1), which is based on measuring the performance in tasks designed to evaluate specific domains of human cognition and their relationship with brain function. While some studies adopting this approach exist in the literature (Carter et al., 2005; Bouso et al., 2013; Pokorny et al., 2020; Healy, 2021), they are underrepresented in comparison to those seeking to determine the neural correlates of self-reported subjective effects. Impaired attention also limits the use of paradigms from psychophysics, as well as other approaches where the main outcome of the experiment is a metric of behavioral performance in an attention-demanding task. Perhaps the most widely adopted approach for the study of psychedelic compounds in humans is that of self-reported questionnaires (right upper quadrant in Figure 1), including versions that can be completed even when undergoing intense effects, such as visual analogue scales (VAS). Over the past decade, an ample variety of questionnaires has been proposed to investigate how psychedelics affect cognition, conscious perception, thought processes, as well as beliefs, attitudes, and personality traits, and their results have been correlated with objective measurements of brain activity obtained using neuroimaging techniques such as functional magnetic resonance imaging (fMRI), electroencephalography (EEG) and magnetoencephalography (MEG) (Dittrich, 1998; Studerus et al., 2010; Barrett et al., 2015; Carhart-Harris et al., 2012; Muthukumaraswamy et al., 2013; Kometer et al., 2015; Carhart-Harris et al., 2016; Barrett, et al., 2016; Nour et al., 2016; Preller et al., 2018; Kettner et al., 2019; Roseman et al., 2019; Timmermann et al., 2019; Pallavicini et al., 2020).

In comparison to paradigms based on constrained tasks and self-reported measures assessed via questionnaires, the study of unconstrained speech produced either spontaneously or in response to simple queries (left upper quadrant in Figure 1) is uncommon, in part due to the difficulty of extracting meaningful objective and quantitative data from unstructured text data. When assessing the merits of verbal or written reports of subjective experiences, this limitation should also be considered a potential advantage, since the range of information that can be gathered by a questionnaire is limited by the choice of questions, while the completeness of subjective reports communicated via natural language is limited by the participants’ capacities for recall and introspection (however, other potential confounds exist–see “Limitations and future directions”) (Kjell et al., 2019). To further ensure the completeness of reports provided in natural language, guided interviews can help individuals to narrate their memories of the experience in an orderly and structured fashion, with techniques such as the microphenomenological interview presenting potential to accurately and reliably capture subjective experience with a level of detail beyond the scope of questionnaires or ordinary natural language reports (Petitmengin et al., 2019). Another advantage of unconstrained reports is their ubiquity. After Aldous Huxley’s famous mescaline “trip report” in “The Doors of Perception” (Huxley, 1954), the practice of retrospectively narrating psychedelic experience steadily increased in popularity, eventually leading to large online repositories of such reports. A prominent example is found in Erowid’s Experience Vaults (https://erowid.org/experiences/), which contain thousands of curated reports of drug-induced experiences across a wide range of chemicals and their combinations. While the usefulness of this data is hindered by several unknowns (which will be discussed later in this review), it nevertheless represents one of the largest sources of information concerning the acute effects of psychedelic drugs. Efforts are underway to systematize the results of psychometric questionnaires available in the scientific literature (such as the Altered States database, http://www.asdb.info/; Schmidt and Berkemeyer, 2018), yet the amount of processed data is still small relative to the number of reports contained in the Erowid database.

**FIGURE 2:**
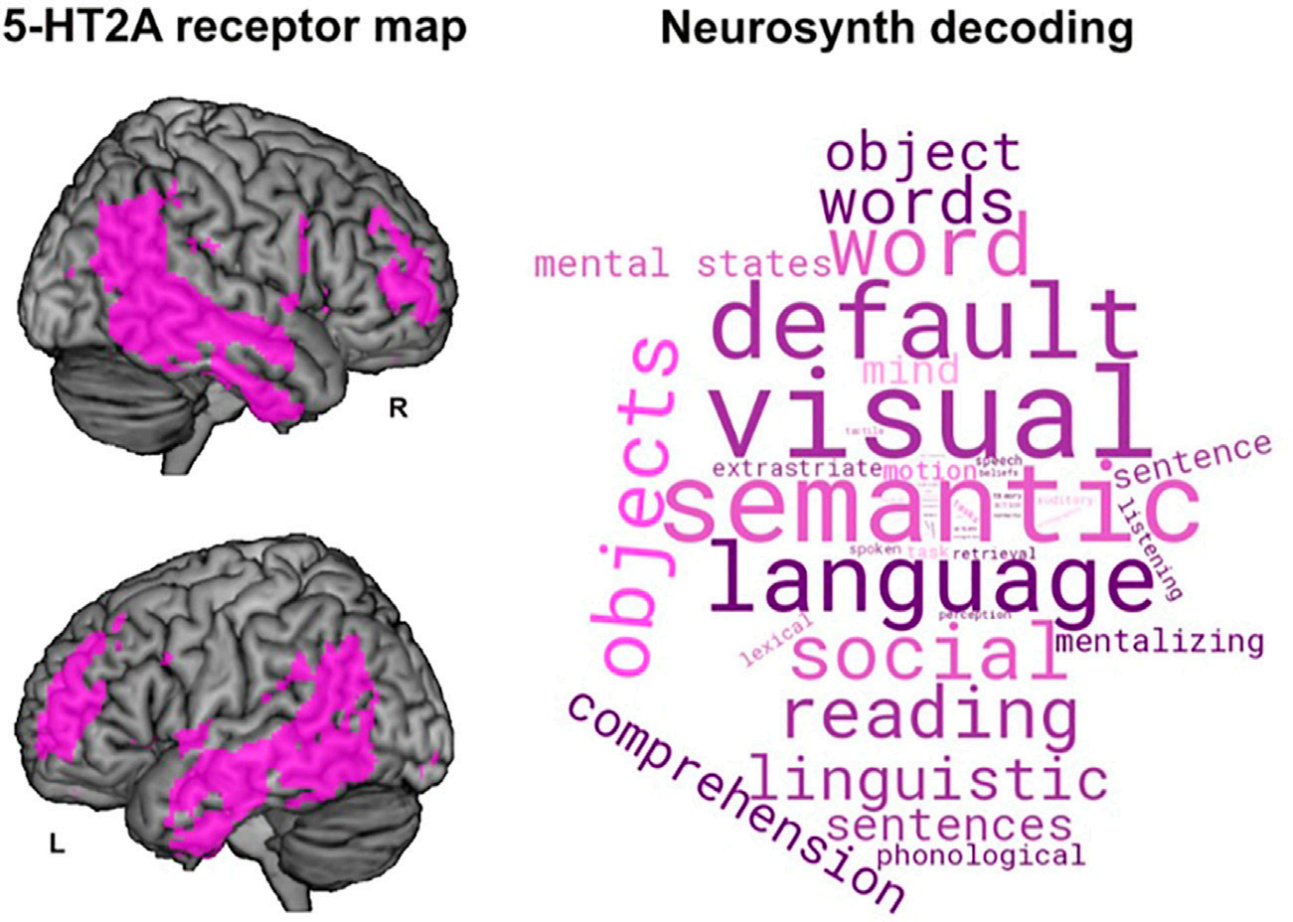
Overlap between regions presenting a high density of 5-HT2A receptors and those consistently involved in language understanding and production, as determined by Neurosynth meta-analytic activation maps (Yarkoni et al., 2011). The 5-HT2A receptor density map (left) was obtained from 95 healthy subjects using Positron Emission Tomography (PET) with [^18^F]altanserin as a radioligand (Saulin et al., 2012). The word cloud on the right was obtained using Neurosynth’s decoder tool applied to the 5-HT2A receptor density map (https://neurosynth.org/decode/). Only terms associated with human cognitive functions were retained in the word cloud; those of neuroanatomical origin (e.g., cingulate cortex, insula) were discarded.

Natural language reports obtained *during* the acute effects of psychedelics open a new dimension of analysis beyond the possibilities of psychometric questionnaires, given by the effects of the drugs on language itself (as opposed to their effects on the semantic content of the reports) (Zilles et al., 2015). As shown in Figure 2, the distribution of regions with high density of 5-HT2A receptors (the main pharmacological target of psychedelics; Nichols, 2016; Zilles et al., 2015) presents substantial overlap with brain activations elicited in neuroimaging experiments of language production and processing. The word cloud on the right of Figure 2 contains terms whose associated meta-analytic activation maps (obtained using Neurosynth, https://neurosynth.org/; Yarkoni et al., 2011) match the 5-HT2A receptor density map; notably, these terms include “semantic”, “language”, “words”, and “reading”, among others related to language. This suggests that 5-HT2A receptor activation can exert a direct modulatory function on the production and understanding of language. In this case, a psychedelic drug could modify certain features of language produced by an individual under its acute effects, including both semantic and non-semantic features. The content of subjective reports constitutes the *message*, while features related to the general structure of spoken language (regardless of the semantics) pertain to the *medium*, which is difficult to investigate solely using questionnaires. As we will discuss in this review, increasing evidence supports that certain language alterations are characteristic of specific neuropsychiatric disorders, which in turn could arise from neurochemical alterations at the level of networks of neurons (Elvevåg et al., 2007; Bedi et al., 2015; Corcoran et al., 2018; Iter et al., 2018; Marggraf et al., 2018; Spencer et al., 2021). We will then argue that relating this body of research with the speech abnormalities observed under the effects of psychedelics could be informative to clarify their mechanism of action, as well as to inform some of their potential therapeutic uses.

## Fundamentals of Natural Language processing

For natural language reports to be useful in the investigation of the phenomenology of psychedelic drugs, a necessary first step is to extract from them reliable and quantitative information. Since these reports are unstructured and unconstrained, their analysis is more challenging than that of questionnaires, for which standardized numerical outcomes are readily available. We distinguish between the analysis of transcripts, focusing only on the text associated with a report, and the analysis of the acoustic features of spoken language. In this review, we will discuss only studies based on NLP applied to transcripts, since the acoustic analysis of drug-induced alterations to speech is almost unexplored in the literature. Nevertheless, we note that this direction of research is promising and should be explored in the future.

The analysis of written reports (or speech transcripts) can be further subdivided into two domains. Semantic analyses are concerned with inferring the meaning intended by the subjects, while non-semantic analyses refer to other aspects of language that are, in general, independent of meaning; for instance, the relative frequency of different parts of speech (noun, pronoun, verb, adjective, adverb, preposition, conjunction, interjection), or the repetition of words or sequences of words, independently of the content they convey.

In the context of this review, the interpretation of *meaning* is based on the distributional hypothesis of semantics (Sahlgren, 2006), stating that the similarity between the meaning of two words depends on their statistical co-occurrence in similar linguistic contexts (as expressed by Wittgenstein, “...the meaning of a word is its use in the language”) (Wittgenstein, 1953). Following this idea, two documents share semantic content if they contain similar words that also appear with similar frequencies. Defined in this way, the semantic similarity depends on the linguistic context used to estimate word co-occurrence; for instance, we expect that for New Yorkers, the semantic similarity between nouns “yellow” and “taxi” is higher than for Londoners, for whom taxis are frequently black instead of yellow. Interestingly, it has been shown that the similarity of neural representations of words correlates with semantic similarity (Carota et al., 2021). Computational analyses suggest that semantically similar words could elicit similar neural representations by increasing the efficacy of synapses through a process of Hebbian learning (Li et al., 2004), although neurobiological support for this possibility has not yet been obtained.

Commonly, texts are first pre-processed to transform words to lemmas (i.e. lemmatization) to reduce the proliferation of different but semantically related terms, such as the different tenses of the same verb. Other common preprocessing steps include removal of very rare or very frequent terms, including stopwords (“a”, “the”, “is”, “are”, etc.). Semantic analyses usually start from a representation of the data given by a term-document matrix, where rows correspond to the terms retained after preprocessing, and documents represent a collection of drug-induced subjective reports. The information about the terms and their use is included in this matrix, which contains in its i,j entry the (normalized) count of the *i*th word in the *j*th document^1^. In principle, computing the similarity (e.g., cosine similarity, linear correlation, etc.) between the rows or columns of this matrix results in a measure of semantic similarity between the terms or documents, respectively. In practice, however, this direct approach is replaced by more sophisticated NLP methods capable of sorting two important obstacles (Landauer et al., 1998). First, if the vocabulary of terms is large, the entries of the matrix will be sparse, i.e. most of the entries will be zero. Similarity measures computed between rows and columns of a very sparse term-document matrix are likely to yield erroneous or misleading results. Second, the direct analysis of the term-document matrix results in semantic similarity estimates based on term co-occurrence between documents; however, two terms might never co-occur in the documents, but at the same time frequently occur together with a third term, thus being semantically related. For example, the number of words shared between the sentences “the garden was full of roses” and “a vase with daisies sits on the table” is zero; however, both sentences are clearly related to the concept of “flowers”, even if that word does not directly appear in them.

**FIGURE 3:**
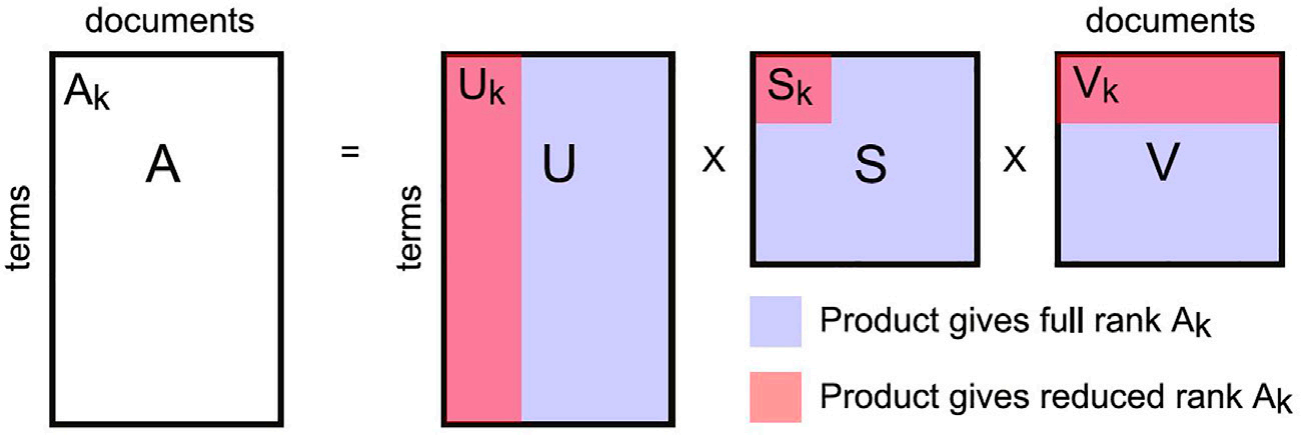
Matrix factorization for LSA. The normalized term-document matrix A can be expressed as the product of three matrices (U, S, V). To reduce the rank of matrix A (and hence alleviate the issues of sparsity and neglect of higher order term co-occurrences) the product can be re-computed retaining only the k principal submatrix of S (thus restricting U and V to their first k columns and rows, respectively). Parameter k is related to the estimated number of latent semantic dimensions in the corpus (Landauer et al., 1998).

Latent semantic analysis (LSA) is a method that simultaneously solves these two problems by reducing the number of linearly independent rows in the term-document matrix, i.e. by lowering its *rank* (Landauer et al., 1998). This results in a less sparse representation where terms can have non-zero similarity even if they never co-occur through the documents (the same in the case of two documents whose words do not overlap). Afterwards, the similarity between pairs of documents can be estimated by computing the cosine distance or the correlation coefficient between the corresponding columns of the term-document matrix. LSA is based on a matrix algebra procedure known as singular value decomposition (Klema and Laub, 1980), a generalization of the eigenvalue decomposition used to factor a rectangular matrix as A = USV, where S is a square diagonal matrix with diagonal entries known as singular values. The number of non-zero singular values is the rank of matrix A. To reduce the rank of A to rank k, the singular value decomposition can be replaced by A_k_ = U_k_S_k_V_k_, with A_k_ the reduced rank matrix, S_k_ the square submatrix obtained by truncating matrix S to keep only the k largest singular values, and matrices U_k_ and V_k_ keeping only the first k columns and rows of matrices U and V, respectively. This procedure is illustrated in Figure 3.

The semantic similarity between words can also be estimated using a family of methods known as word embeddings. These methods can be used to map the vocabulary into a vector space of reduced dimensionality, with proximity within the vector space indicating semantic similarity and vice-versa. Methods such as word2vec are capable of learning this vector space from data using a shallow neural network trained to infer the words surrounding each term in the training vocabulary (Mikolov et al., 2013). Both LSA and word embeddings are behind some of the most promising NLP-based markers of altered thought processes in psychiatric patients; for instance, they can be used to define metrics of semantic coherence, i.e. the extent to which the semantic content of speech changes unexpectedly, instead of following a more continuous and predictable trajectory (Elvevåg et al., 2007; Bedi et al., 2015; Corcoran et al., 2018; Iter et al., 2018; Marggraf et al., 2018; Spencer et al., 2021).

In contrast, non-semantic methods treat written texts or speech transcripts as sequences of tokens arranged according to syntactical rules, regardless of their meaning. Along this line, the method of speech graphs transforms texts into graphs or networks, where nodes correspond to words (before or after lemmatization) and the directed links indicate that one word follows another in the text. The topological properties of the resulting graphs contain information useful to characterize drug-induced language alterations, as well as abnormalities specific to certain neuropsychiatric disorders (Mota et al., 2012; Bertola et al., 2014; Mota et al., 2014; Mota et al., 2017; Coelho et al., 2021). Some examples of these topological properties include the number of cycles of different length, the average shortest path in the graph, the maximum distance across all pairs of nodes (diameter), among others (Bullmore and Sporns, 2009).

As a general observation, semantic methods are useful to investigate retrospective subjective reports in terms of their content (for instance, what happened during the experience, how it felt to the subject, etc.) and to compare the experiences narrated in different reports in terms of their shared content. Non-semantic methods applied to these reports are unlikely to provide information beyond the individual traits or writing styles of the participants. However, non-semantic analyses are useful to determine how the overall structure of verbal expression is modified during the acute effects (the medium), regardless of the particular content that is being expressed (the message), which could inform the pharmacological modulation of language production networks in the brain.

## Language Production Under the Acute Effects of Psychedelics

Among their many potential uses and denominations, serotonergic psychedelics have been called “psychotomimetics” (meaning “psychosis-mimicking”) and investigated as agents capable of eliciting states of altered consciousness and cognition that are similar to psychosis (Nichols and Walter, 2020). Over the last decades, the analysis of speech production has shown great promise to detect and predict psychotic episodes; in particular, many these studies have found that the discourse of psychotic patients tends to show reduced semantic coherence in comparison to that produced by healthy individuals (Wykes and Leff, 1982; Fraser et al., 1986; Hotchkiss and Harvey, 1986; DeLisi, 2001; Covington et al., 2005; Elvevåg et al., 2007; Bedi et al., 2015; Corcoran et al., 2018; Iter et al., 2018; Marggraf et al., 2018; Spencer et al., 2021). Consistent with their purported role as psychotomimetics, early studies demonstrated that psychedelics also render speech less predictable and enhance free-association (Amarel and Cheek, 1965), while a more recent study based on a picture naming task showed that LSD enhances semantic associativity (Family et al., 2016). Another recent work used psychometric questionnaires and language processing tasks to show that LSD affects the stream of thought at multiple levels, facets, and time points after infusion (Wießner et al., 2021). However, the characteristics of unconstrained speech produced during the acute effects of psychedelics remain relatively underexplored from the perspective of NLP, an approach that is becoming increasingly commonplace in computational psychiatry (Abbe et al., 2016; Le Glaz et al., 2021).

**FIGURE 4:**
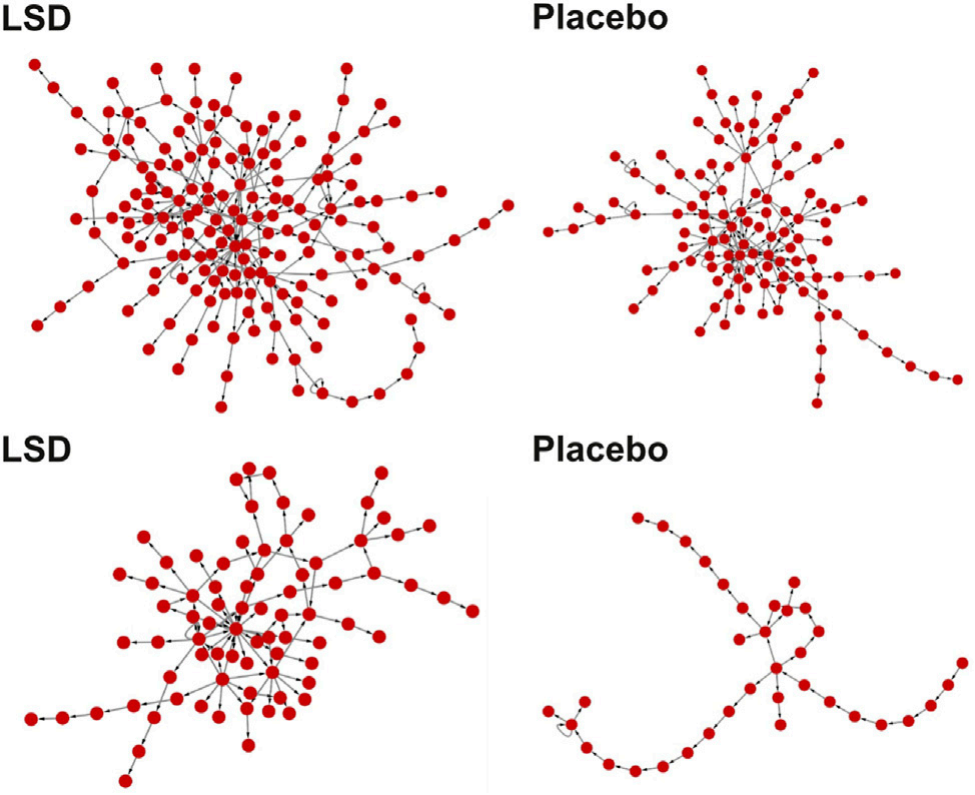
Alterations in non-semantic features of speech produced under the acute effects of 75 μg of LSD. Example graphs encoding speech produced under the acute effects of LSD (left) and under a placebo (right). Each node (red circles) corresponds to a word in the transcripts, and the directed links indicate that one word (target node) followed another (source node) at least once during the speech transcript (Sanz et al., 2021).

An exception can be found in recent work by Sanz and colleagues, where authors applied semantic and non-semantic methods to interviews conducted at two different time points after intravenous infusion of 75 μg of LSD (Sanz et al., 2021). Semantic coherence (computed as the variance of the distance between the word2vec embeddings of pairs of subsequent words) was reduced during the acute effects of the drug, while speech graphs presented a more recurrent structure, with increased verbosity and decreased lexicon. Example speech graphs for LSD and placebo are shown in Figure 4, where the scrambling effects of LSD on the organization of natural language can be directly appreciated. Finally, LSD increased Shannon’s entropy, which represents the average rate at which information is produced by the participants (Shannon, 1948).

These changes are generally consistent with the psychotomimetic hypothesis; however, while both schizophrenic and manic psychosis are linked to reduced speech coherence, only the latter presents more recurrent speech graph structure with increased verbosity (Mota et al., 2012). Sanz et al. analyzed reference samples from bipolar patients to demonstrate the similarity with LSD-induced acute changes in speech. The entropic brain hypothesis put forward by Carhart-Harris and colleagues is a more general framework to interpret these findings (Carhart-Harris et al., 2014; Carhart-Harris, 2018). According to Carhart-Harris and colleagues, 5-HT2A receptor stimulation by psychedelics has a net scrambling or disorganizing effect on brain activity at multiple spatial and temporal scales, including those associated with perception, cognition, and the production of language. Conversely, antipsychotic medications show effects opposite to those predicted by the entropic brain hypothesis, leading to slower articulation rate, increased pausing, shorter utterances, and overall less information production rate (de Boer et al., 2020). These changes in language production are related to D2 receptor antagonism, the main pharmacological mechanism of action of antipsychotic drugs; however, future studies should assess the effects of more specific 5-HT2A receptor antagonists (e.g., ketanserin) on non-semantic language features in the light of the entropic brain hypothesis. Also, Sanz et al. investigated LSD, a drug presenting biphasic effects with a gradual shift from serotonergic to dopaminergic action. Given that some of the strongest effects were seen close to the second interview (225 min post-infusion), a role of dopaminergic stimulation on speech disorganization cannot be ruled out (Marona-Lewicka and Nichols, 2007).

There is an interesting discrepancy between the results published by Sanz and colleagues and early studies of speech under the effects of LSD, which seemed to favor similarities between schizophrenic psychoses and the psychedelic state. Amarel and Cheek (1965) showed that LSD reduced the predictability of speech by means of impaired semantic coherence, but also reduced the total number of spoken words. It must be noted, however, that Amarel and Cheek investigated individuals suffering from alcoholism in a clinical setting, a population that could have experienced high levels of unattended anxiety during the experience. In contrast, Sanz et al. investigated healthy and motivated subjects with significant previous experience with psychedelic drugs, who willingly decided to participate in the experiment. As usual with psychedelics, the importance of context (i.e. the set and setting) in the interpretation of experimental results should be not underestimated (Carhart-Harris et al., 2018).

Natural speech produced during or immediately after the acute effects of psychedelics can also be used to compare 5-HT2A receptor activation with other altered states of consciousness, such as rapid eye movement (REM) sleep, the part of sleep characterized by long and vivid dream reports after awakening. Kraehenmann and colleagues instructed their subjects to produce verbal reports of a guided mental imagery task conducted 7 h after treatment with LSD or a placebo (Kraehenmann et al., 2017). Afterwards, they manually scored the cognitive bizarreness of reports produced under both conditions, and found it was significantly increased for the LSD condition. High cognitive bizarreness is typical of dream reports, being indicative of the “strange, irrational and fanciful quality of REM sleep dream mentation, which is characterized by the presence of improbable or impossible imaginary events, characters, objects, thoughts or feelings” (Hobson et al., 1987; Kraehenmann et al., 2017); thus, its increase is consistent with the loss of semantic coherence reported by Sanz and colleagues.

Lastly, alterations in the production of language could be characteristic of drugs that affect other neurotransmitter systems, given that language-related cortical areas are characterized by specific neurotransmitter receptor fingerprints (Zilles et al., 2015). Some examples are given by changes in the use of action-related verbs related to the dopaminergic system (Norel et al., 2018), and by the induction of formal thought disorder by ketamine, resulting in functional lateralization during speech production (Nagels et al., 2018). Thus, the analysis of natural language could be useful to characterize the effects and mechanism of action of novel psychoactive drugs, including psychedelic compounds with affinity for a wide range of receptors besides 5-HT2A (Ray, 2010).

## Analysis of Retrospective Reports

Before the development of psychometric questionnaires, spoken or written retrospective reports constituted the principal medium to communicate the nature of different drug-induced experiences. We find a well-known example in the following passage from “The Doors of Perception”, by Aldous Huxley:

“Like the flowers, they glowed, when I looked at them, with brighter colors, a profounder significance. Red books, like rubies; emerald books; books bound in white jade; books of agate; of aquamarine, of yellow topaz; lapis lazuli books whose color was so intense, so intrinsically meaningful, that they seemed to be on the point of leaving the shelves to thrust themselves more insistently on my attention” (Huxley, 1954).

After returning to baseline, the author describes the effects of the drug, recalling in this case how certain aspects of visual perception (the colors and salience of books in shelves) were profoundly altered. We note that the semantic content of this retrospective subjective report constitutes a first approximation to the subjective effects elicited by the drug; thus, if the author wrote about altered color perception, dizziness and anxiety, we can suspect that the experience included at least some of these effects. Moreover, if two sets of reports stemming from the use of different drugs are similar in terms of their semantic content, we can hypothesize that the drug-induced experiences were similar as well. In other words, the semantic similarity of retrospective reports could be used as a measure of similarity between the reported experiences.

This approach was first applied by Coyle et al. to the corpus of the Erowid Experience Vaults (Coyle et al., 2012). This corpus contains reports for an ample variety of psychoactive compounds; also, some of the compounds are linked to a very large number of reports. Coyle and colleagues used supervised machine learning methods to successfully separate between reports of different drugs based on the associated vocabulary frequency vectors, showing that different families of compounds were linked to narratives with distinctive semantic content. Importantly, the preprocessing of the data included standard steps, such as removal of stopwords and lemmatization, but also the identification and removal of drug-specific terms and of terms that are generally related to drug use, since these features could allow a highly accurate yet trivial classification of the reports.

**FIGURE 5:**
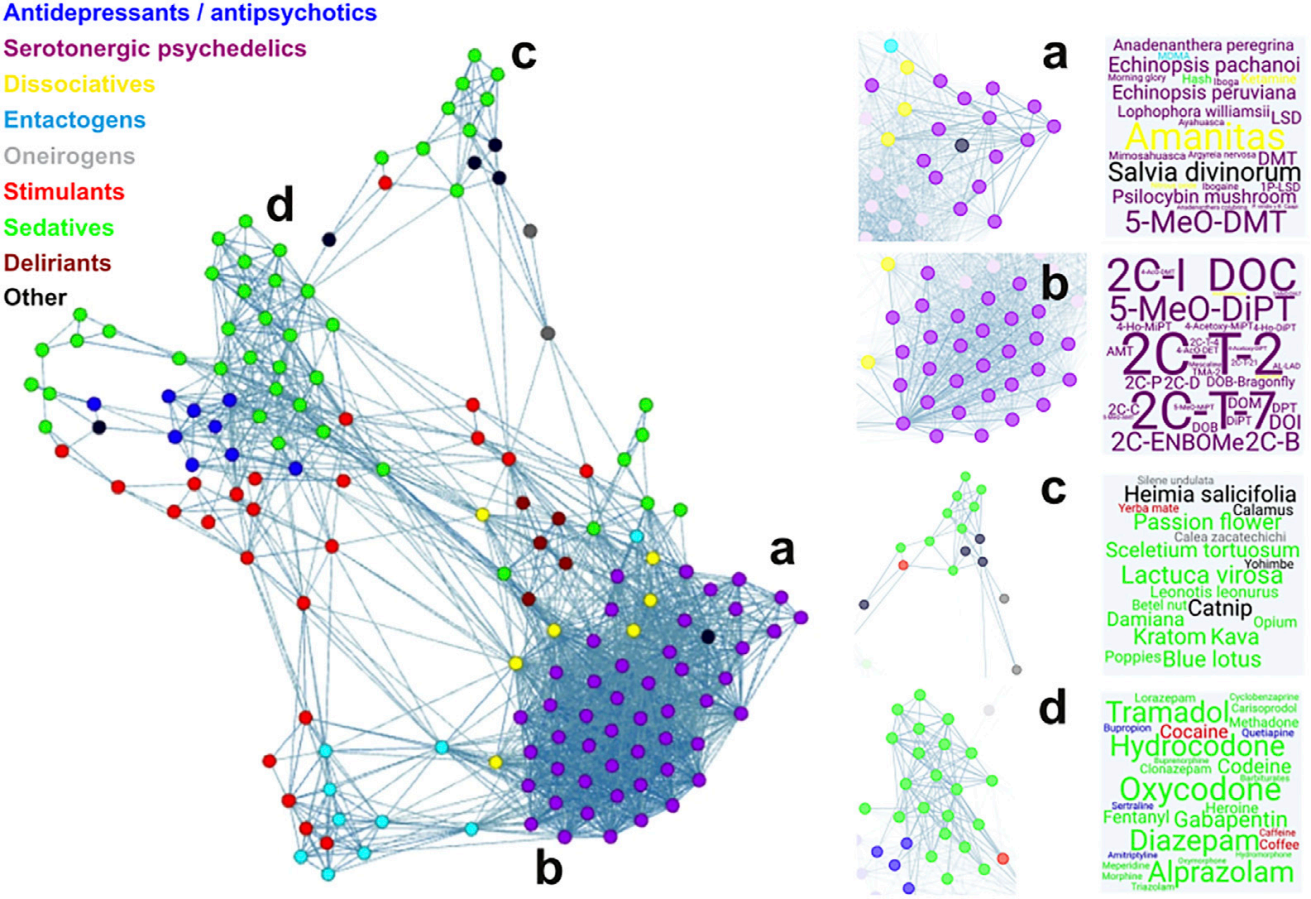
Semantic similarity network for reports of drug-induced experience (Sanz et al., 2018). Each node represents the combined Erowid’s reports for a certain drug, colored according to categories based on their known effects and mechanism of action (see labels in the upper left). A connection between a pair of nodes indicates that the corresponding pair of reports present a sufficiently high degree of semantic similarity. The network was drawn using the ForceAtlas layout included in Gephi (https://gephi.org/), which tends to separate and identify groups of highly interconnected nodes that have sparser connections with the rest of the network (known as *modules*). Subpanels highlight different network modules, and the associated word clouds contain the name of drugs belonging to each module, with size weighted by their average strength of within module connections. These modules correspond to mostly natural and synthetic psychedelics (panels **(A,B)**, respectively), and to mostly natural and synthetic sedatives (panels **(C,D)**, respectively).

Sanz and colleagues applied a similar yet more sophisticated framework to investigate the relationship between the pharmacological mechanism of action of a widespread variety of drugs and the shared semantic content of their associated Erowid reports (Sanz et al., 2018). This approach included the preprocessing steps already implemented by Coyle et al., but then used LSA to tackle the issue of sparse term-document matrices. Figure 5, based on their analysis, contains the unsupervised classification of several drugs based on the semantic similarity of their associated subjective reports. Briefly, the reduced-rank term-frequency matrix reconstructed using LSA was used to compute the correlation matrix C_ij_, which has in its i,j entry the correlation between the term frequency vectors of the *i*th and *j*th drugs in the corpus. This matrix was then used as the input of an unsupervised module detection algorithm (Blondel et al., 2008) capable of identifying drugs whose within-group semantic similarity was higher than their similarity with other similarly identified groups (note that the network representation in Figure 5 only shows links between drugs whose reports have a high semantic similarity). These modules of similar drugs (in the semantic sense) recapitulate known subjective effects and mechanisms of action; for instance, it is possible to find modules composed mostly of natural (module a) and synthetic (module b) psychedelic compounds. The same happens for modules composed of sedative drugs (modules c and d). Overall, the unsupervised classification of drugs in terms of the semantic content respects traditional categories such as antidepressants and antipsychotics (most commonly, prescription drugs), psychedelics, dissociatives, entactogens, stimulants, and sedatives, among others.

Interestingly, the comparison of subjective effects by means of retrospective reports is not limited to drug-induced experiences. As shown in Sanz et al. (2018), it is possible to construct a ranking of drugs in terms of the semantic similarity between their Erowid reports and dream reports, i.e. narratives about the content of individual dreams. Dreaming during sleep is a non-ordinary state of consciousness characterized by vivid multimodal imagery (ranging from elementary percepts to full-blown immersive hallucinations), altered sense of the relationship between the self and body boundaries, loss of the sense of agency, suppressed metacognitive function and heightened emotional reactivity (Nir and Tononi, 2010). According to anecdotal reports, these effects partially overlap with those elicited by psychedelic drugs, which has led several authors to speculate about a possible relationship between both states; however, the evidence directly supporting this overlap of effects is scarce (Snyder, 2001; Hobson, 2002; Carhart-Harris and Nutt, 2014; Kraehenmann, 2017). According to the large-scale analysis conducted in Sanz et al. (2018), psychedelics are the closest to dreams of high lucidity (i.e., when the dreamer is aware of being in the dreaming state; Voss et al., 2009) in terms of their induced subjective experience, with LSD being the closest across all drugs in the Erowid corpus. Consistently with their known subjective effects (Krenzelok, 2010), experiences induced by deliriant compounds (e.g., those found in plants of the Datura genus) are the most similar to dreams of low lucidity. The use of LSA applied to retrospective subjective reports was also employed to demonstrate that ketamine, a glutamatergic dissociative agent, has subjective effects most similar to those reported after “near death experiences”, i.e., episodes with real or perceived proximity to death that reliably include feelings of disconnection with the body (out of the body experiences) and of floating towards a light through a tunnel, a profound sense of bliss, a fast review of autobiographical memories, and the sense of an irreversible threshold (Martial et al., 2019). Interestingly, the purported neuroprotective properties of ketamine (more precisely, its capacity to prevent cell death through over-excitation by antagonism at N-methyl-d-aspartate [NMDA] receptors; Hudetz and Pagel, 2010) led to speculations of an endogenous chemical with a similar mechanism of action that is released at times when neuroprotection could help to increase the likelihood of survival (e.g., hypoxia) (Jansen, 1997). However, the acute effects of ketamine and related dissociative agents might negatively affect the survival of the organism when faced with situations that require a rapid and precise response (although a possible evolutionary foundation of near-death experiences is thanatosis, i.e., death feigning behavior) (Peinkhofer et al., 2021). Moreover, near death experiences have been reported by individuals who did not suffer major physical trauma, which casts further doubt on the neuropharmacological origin of these experiences (Charland-Verville et al., 2014).

These studies foreshadow a possible research program to pursue a taxonomy of conscious states based on data-driven similarity metrics, in particular, those constructed using natural language reports of subjective experiences. Either implicitly or explicitly, most researchers operate under the assumption that consciousness can be graded along a unidimensional continuum, leading to the notion of “levels of consciousness”. For instance, according to this idea, states such as coma or general anesthesia are “less conscious” than light sleep, which in turn is “less conscious” than ordinary wakefulness (Laureys, 2005). Some of the conceptual problems with this notion have been highlighted by Bayne and colleagues (Bayne et al., 2016); in particular, the acute effects of serotonergic psychedelics are especially hard to reconcile with the idea of levels of consciousness (Bayne and Carter, 2018). While some popular accounts endorse the notion of the psychedelic state as an “expanded” (as opposed to diminished) state of consciousness, in reality psychedelics severely impair some aspects of human cognition and perception, while sparing or even facilitating others (Carter et al., 2005; Bouso et al., 2013; Bayne and Carter, 2018; Pokorny et al., 2020; Healy, 2021). Taking these considerations into account, several independent dimensions are likely to be required to fully characterize the repertoire of conscious states, yet disagreements exist about what these dimensions should be (Bayne et al., 2016; Bayne and Carter, 2018; Fortier-Davy and Millière, 2020).

The type of analysis represented in Figure 5 might represent an interim solution to this open problem: it is not necessary to find the dimensions needed for the adequate description and characterization of conscious states if one is capable of estimating their pairwise similarity. Crucially, this similarity could be estimated in terms of the shared semantic content of natural language reports. While the similarity between the subjective experience of different conscious states could be inferred from the comparison between psychometric questionnaires, without knowing the relevant dimensions necessary to capture the variability of states it does not seem possible to design the adequate questionnaires in the first place. The computational analysis of retrospective narratives seems to offer a data-driven alternative to this conundrum, since it allows to estimate the similarity of effects based on the semantic distance between reports, without constraining the participants to answer a predefined set of questions.

## Semantic Similarity Parallels Neurochemical and Pharmacological Similarity

What is the relationship between the mechanism of action of different drugs and the subjective effects they elicit? This is perhaps one of the most difficult yet fascinating questions in consciousness research. Drug-induced experiences are well-characterized at the molecular and cellular levels; for instance, radioligand-binding experiments in which a test ligand competes with a high-affinity radiolabeled ligand allow the *in vitro* estimation of drug affinities for specific binding sites, which include most of the major neurotransmitter receptors types and sub-types (Allen and Roth, 2011). This information can be summarized in drug-specific affinity profiles. Also, for certain drugs and receptors much more information is available, including functional assays (informing whether the drug behaves as an agonist, antagonist, or inverse agonist), changes in gene expression (González-Maeso et al., 2003), and second messenger recruitment in G-protein coupled receptors (López-Giménez and González-Maeso, 2017). However, in spite of this detailed knowledge at the molecular scale, comparatively less is known about the downstream effects on large-scale activity patterns that correlate with cognition and conscious experience.

**FIGURE 6:**
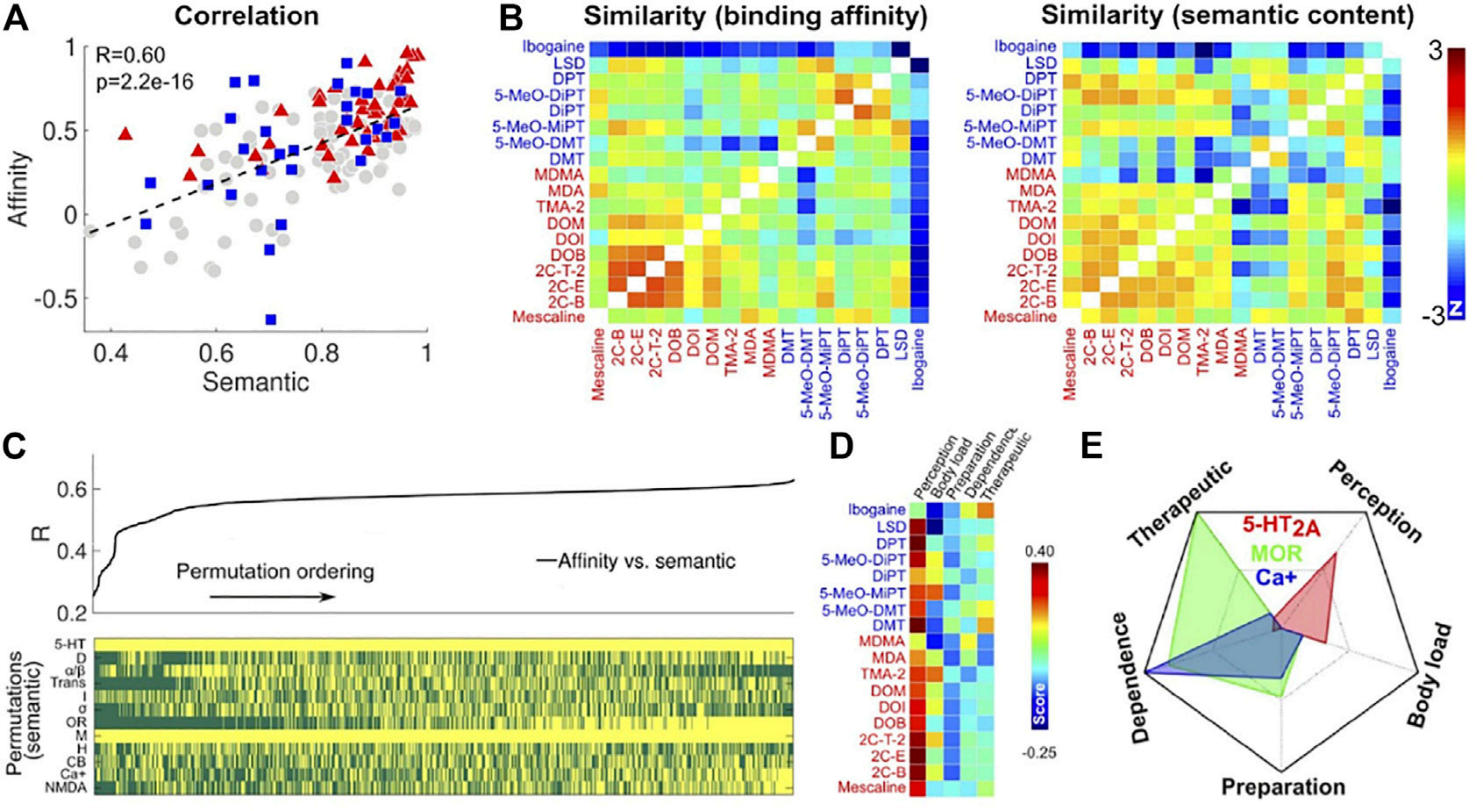
The semantic similarity of subjective reports is correlated with the similarity of binding affinity profiles for 18 psychedelic tryptamines and phenethylamines (panel **(A)**, left), from Zamberlan et al., 2018. This can be appreciated by direct visual inspection of the corresponding similarity matrices (panel **(A)**, right). Panel **(B)** shows the correlation (with the color code representing Z scored correlations) between semantic and pharmacological similarity matrices, with the latter computed using information from different receptor subsets, allowing to identify an optimal group of receptors to explain the semantic content of the reports. The principal components of the reduced ranked term-document matrix represent recurrent topics in the corpus, with variable prevalence among the psychedelic drugs under study (panel **(D)**). In this panel, the color code indicates the load of each LSA topic in the reports associated with each compound. This type of analysis is also capable of linking affinity to certain receptors with specific topics (panel **(E)**), e.g., 5-HT2A with the “perception” topic, or MOR with the “dependence” and “therapeutic” topics (5-HT2A: serotonin 2A receptor, MOR: opioid receptor, Ca+: Calcium ion channel).

Recent work by Zamberlan and colleagues pursued the following hypothesis: if the subjective experience of psychedelic drugs is determined by their action at the molecular level, then the closest two drugs are at this level (e.g. the most similar their binding affinity profiles are), then the closest those drugs should be in terms of the semantic content of their associated subjective reports (Zamberlan et al., 2018). The scatter plot presented in Figure 6A provides support for this hypothesis, showing a positive (although modest) correlation (R = 0.6) between pairwise semantic similarity and the similarity of binding affinity profiles (compare also the binding affinity and semantic similarity matrices in the same panel). While not perfect, this correlation is particularly interesting given it was obtained from a group of drugs that were, for the most part, serotonergic psychedelics (Ray, 2010). It is well established that these compounds exert their effects primarily by (partial) agonism at 5-HT2A receptors (Nichols, 2016), yet if this is the only relevant mechanism of action, how can different psychedelic drugs present a variety of different effects? A candidate solution is explored by Zamberlan and colleagues: while 5-HT2A agonism is a necessary condition for psychedelic activity, the action of the drug at other binding sites can nuance the resulting subjective effects (note that this hypothesis is not mutually exclusive with the possibility of functional selectivity at GPCR, such as the 5-HT2A receptor; Nichols, 2017).

The relationship between subjective effects and neurochemical action of psychedelic compounds can be further informed by natural language processing applied to subjective reports. Suppose the binding affinity similarity between two compounds is not computed by comparing their affinities across all assayed receptors, but instead restricted to a subset of binding sites. In this case, if the correlation between binding affinity and semantic similarities increases, it could be concluded that activity at this subset of receptors is more specific to determine the subjective effects of the compound. This idea is exploited in the analysis shown in Figure 6C, showing the correlation between semantic and affinity similarities computed using the binding affinity at several possible combinations of receptor subsets. The top subpanel of Figure 6C, extracted from Zamberlan et al. (2018), shows the obtained correlation values, sorted in ascending order, while the lower panel highlights in yellow whether the receptor families in the rows were considered for the computation of the binding affinity similarities (these receptor families include serotonergic, dopaminergic, adrenergic, histaminergic, glutamatergic and cannabinoid receptors, plus transporter proteins and Ca+ channels, among other groups of binding sites). In principle, this approach could be inverted to specify combinations of receptors to be targeted with the objective of eliciting certain subjective effects.

Taking this analysis one step further, it is possible to decompose the subjective reports into topics, given by sets of terms that tend to co-occur through the documents, summarizing certain repeating themes. Figure 6D lists some topics obtained by Zamberlan and colleagues using the reports associated with several psychedelic tryptamines and phenethylamines, which include topics such as “changes to visual perception”, “body load”, “drug preparation”, “drug dependency” and “therapeutic use”; this panel also presents the prevalence of each topic (columns) across the drugs (rows). By relating topics and affinities it is possible to inform the subjective effects linked to high binding affinity at different sites, as shown in Figure 6E for three example receptors (note that high affinity for 5-HT2A indicates the prevalence of the “changes to visual perception” topic, as expected). The idea of linking receptor activation with subjective effects through NLP applied to retrospective reports has been leveraged by Ballentine and colleagues to map brain areas whose high density of different neurotransmitter receptors correlates with specific reported subjective experiences (Ballentine et al., 2021). This work related the semantic content of more than 6.000 reports of drug-induced experiences with the binding affinity at 40 neurotransmitter receptor subtypes, which were then mapped to 3D coordinates in the brain via their gene transcription levels from invasive tissue probes. While transcriptomic data is only an indirect marker of receptor protein expression, these results could be refined as more *in vivo* PET imaging data becomes available and is curated for integration with structural and functional brain images (Hansen et al., 2022).

## Natural Language Reports and the Therapeutic Use of Psychedelics

After decades of little to no research, an ongoing surge of studies has demonstrated the potential usefulness of psychedelics to treat psychiatric disorders such as depression and anxiety (Sessa, 2005; Mertens and Preller, 2021; Nutt and Carhart-Harris, 2021; Yaden et al., 2021). In contrast to other treatments, the subjective effects induced by psychedelics appear to influence the outcome of the intervention - for instance, current evidence suggests that patients who undergo mystical-type experiences evolve comparatively better after drug intake (Griffiths et al., 2016). It is important to note that the role of mystical-type experience in psychedelic therapy was brought into question by results suggesting that subjective effects might not play a significant role in the long-lasting changes produced by psychedelic compounds (Olson, 2020). However, reports of subjective experiences could nevertheless be useful to predict the outcome of psychedelic-assisted therapy even in the absence of a causal relationship. Considering the correlation between the occurrence of certain conscious experiences and the therapeutic action of psychedelics, it is reasonable to hypothesize that natural language reports carry relevant information to predict the outcome of interventions using these compounds.

This possibility has been first explored by Carrillo and colleagues, who showed that baseline interview data can predict which patients will respond to psilocybin for treatment-resistant depression (Carrillo et al., 2018). In this study, quantitative metrics related to the emotional content of the interviews were extracted and used as input to train machine learning algorithms, which were then used to identify responders from non-responders with significant (∼80%) accuracy.

The conceptual framework introduced in this study was further developed by Cox and colleagues, and applied it to a much larger dataset of natural language narratives to determine who among more than 1,000 individuals would quit or reduce using drugs following a psychedelic experience (Cox et al., 2021). Here, the features for the machine learning analysis were based on the semantic content of the narratives, more precisely on the prevalence of different topics. While the accuracy was below the value reported by Carrillo and colleagues, it nevertheless reached statistical significance (∼60%), showing that natural language reports obtained from a large sample of individuals have information relevant for the prediction of therapeutic outcomes.

In the same way that NLP could be useful to identify patients who could benefit from psychedelic therapy, it could also be applied to screen for mental health conditions that could present problematic interactions with psychedelics. One example is the detection of individuals at risk of developing psychosis based on machine learning methods applied to linguistic features obtained using NLP (Bedi et al., 2015; Corcoran et al., 2018). This information could be used to further investigate whether certain patients are apt to be treated using psychedelics, as well as to establish precautions to maximize safety and minimize the associated risks.

The use of natural language processing in the incipient field of psychedelic-assisted therapy is not an isolated trend. In recent years it has become evident that the data fingerprint produced by an individual contains information relevant for the early diagnosis and prognosis of several neuropsychiatric disorders (Meyer-Lindenberg, 2018; Sigman et al., 2021). Speech produced under natural or ecological conditions is perhaps among the most useful streams of data that can be tapped for automated machine learning models of mental health, since it is produced in large amounts, it is cheap to obtain and analyze, and might reflect the emotional content of the speakers, as well as their ongoing thought processes (Garfield et al., 1992; Le Glaz et al., 2021). Based on these examples, we can speculate that the automated analysis of natural speech will consolidate into a valuable tool to assist the decision-making process of clinicians, and to optimize the design and implementation of therapy sessions assisted by psychedelic compounds, with the objective of maximizing therapeutic gain and reducing the likelihood of anxiety in the patients. Because of this, we recommend that future clinical studies include the recording of natural speech samples before, during and after the acute effects of psychedelic compounds.

Finally, it is important to discuss the potential ethical implications of developing and implementing NLP-based tools to assist with clinical decision making (Cohen et al., 2014). To date, the accuracy of NLP-based predictive algorithms is comparatively modest, raising the concern of false negatives, i.e. wrongly suggesting to withhold psychedelic therapy from patients who could benefit from it. Also, re-training the algorithms based on their own past predictions could lead to “self-fulfilling prophecies” where predictions of positive outcomes are concentrated in participants with certain characteristics, leading to increased effort in the treatment of these patients, and finally to the reinforcement of the model predictions at the expense of withholding treatment from other groups of patients. These serious issues (which are not limited to NLP-based models) highlight that machine learning tools should not replace human experts, being useful only as additional sources of information to be considered in the process of clinical decision making (Carhart-Harris et al., 2021).

## Limitations and Future Directions

We have reviewed several key studies illustrating how the analysis of written or spoken natural language reports can assist the investigation of the subjective effects and mechanisms of action of psychedelic compounds. The usefulness of this approach, however, must be considered within the context of several limitations, some of them intrinsic to the acquisition and analysis of natural language.

The most obvious limitation of this approach is also related to one of its main potential advantages: the unconstrained nature of natural language reports. When subjects speak freely about a certain subject (for instance, about their subjective experience with a psychedelic compound) they might omit important details or fail to clearly express the most relevant information. Additionally, some subjects might be better communicators than others; their reports are then expected to be more informative in comparison. Since they are participating in a scientific experiment and being interviewed by a specialist, subjects might incorrectly assume that certain information is implicit in the conversation and thus fail to include it in their reports. Moreover, some personality traits of the participants could be manifest in their reports, confounding attempts to objectivize their content using automated methods (Philip et al., 2019). In contrast, standardized psychometric questionnaires have the advantage of uniformly guiding the participants through the points that are considered most relevant by the researchers. Overcoming this limitation depends on training the interviewers to obtain the most of the participants and their reports, for instance, by guiding them through a series of predefined questions, or by bringing them back on track in case they begin to digress. One example of this procedure can be found in the microphenomenological interview (Petitmengin et al., 2019). Interviewers adopting this method are trained to identify the main contents of the narrated experience, to assist the subjects in their evocation process, to detect and document implicit information, and to assist the subjects in the process of formulating their reports, among other precautions.

The analysis of large online databases (e.g., Erowid’s Experience Vaults) presents additional problems associated with unknown or underinformed variables, such as the precise nature of the compounds that were consumed, their dosage, whether drugs were consumed alone or in combination with others, subject demographics, mental health status and past history of drug use. Furthermore, except for relatively recent projects (see for example https://effectindex.com/), ongoing efforts for the collection of drug use reports query participants about their experiences using a single text box, and thus fail to distinguish between preparation, set and setting, the acute phase of the experience, and its short and long term consequences. More adequate interfaces should allow users to explicitly state the precise times when certain events occurred, which are frequently impossible to recover from unstructured reports. While some of these problems could be overcome by imposing more structure to the reports (e.g., forcing participants to narrate separately different aspects of the experience), other limitations (such as insufficient or unreliable information concerning drugs) are more difficult to solve. In those cases, it should be determined whether the availability of large amounts of data can compensate for the potential noise introduced by these limitations.

Other important issues concern the differences between speech produced under the acute effects of psychedelics and after the experience (i.e., retrospective reports), and how the capacity to produce intelligible speech changes across its duration. The comparison between reports produced during and after the experience could be important to reveal systematic changes in how certain experiences are recalled. Indeed, it is known that classic psychedelics dose-dependently impair different aspects of memory, while simultaneously increasing the vividness of affectively intense memories, which raises the possibility of inducing false memories (Healy, 2021). Also, changes in the capacity to produce spoken reports throughout the acute effects of a psychedelic drug could induce systematic sampling biases in the data, overrepresenting certain phases of the experience (and thus certain subjective effects) in the reports. Future studies should address the time course of how psychedelics modulate linguistic capacities, including the period when the effects have completely worn off (e.g., days and weeks after the experience).

In spite of these potential shortcomings and important questions to be answered, the analysis of natural language reports can still be considered a promising tool to tackle research questions about the nature of subjective experience, in particular, those about effects and mechanism of action of serotonergic psychedelics. When finally answered, some of the following questions will serve to more precisely outline the advantages and limitations of natural language over more traditional and established methodologies.

Is it possible to infer the score of psychometric scales, such as 5D-ASC, from unconstrained reports? This question could be answered at the level of individual reports, or at the level of the average scores obtained for a given drug.

Is it possible to accurately estimate the dosage of a drug based on reports obtained during the acute effects, or based on retrospective reports obtained after return to baseline? In general, which of these two approaches conveys more information about this and other facets of the experience?

Can we disentangle the contents of a report from the individual traits of the reporter? How are these traits manifest in a typical report? What is the effect of language, culture, social status, age, gender, etc., on the way participants express their drug-induced experiences?

When used to predict the outcome of psychedelic treatments, how does the accuracy obtained using natural language reports compare with that obtained using questionnaires? In case the accuracy is significantly higher, is it possible to identify the information that is conveyed by natural language reports that tends to be left out of more structured assessments?

How is the semantic similarity between drug use reports related to different metrics of similarity between drugs and their effects on the brain? At which level of description is the semantic content of the reports optimally represented?

What is the temporal resolution of retrospective narratives? How much temporally ordered information about subjective experience can be conveyed by means of natural language reports, and how is this information distorted?

How robust is NLP analysis of subjective reports with respect to the language used to express those reports?

## Conclusion

Language is our main everyday vehicle for the expression of ideas, emotions, plans, and subjective, inner feelings. After experiencing the acute effects of a psychedelic drug, many individuals feel a strong urge to communicate with others and exchange impressions about what happened and how it felt. Taken together, the articles we reviewed serve to demonstrate how this urge can be leveraged for the scientific exploration of serotonergic psychedelics. While the merits of natural language processing over more traditional methods to inquire about subjective experiences remain to be demonstrated, we nevertheless encourage researchers to acquire natural language samples in their experiments, and to address some of the open questions that will ultimately determine the usefulness of this approach.

## Footnotes

1 This can be extended to represent pairs of adjacent words in the matrix (bigrams), or more generally, sequence of n consecutive words (n-grams). However, increasingly larger text corpora are required to adequately sample n-grams as n increases.
